# An optimized swine dysentery murine model to characterize shedding and clinical disease associated with *“Brachyspira hampsonii*” infection

**DOI:** 10.1186/s12917-017-1166-5

**Published:** 2017-08-22

**Authors:** Courtney E. Ek, Roman Nosach, Champika Fernando, Yanyun Huang, Jason Byron D.S. Perez, Matheus O. Costa, Samantha Ekanayake, Janet E. Hill, John C.S. Harding

**Affiliations:** 1 Department of Large Animal Clinical Studies, Saskatoon, SK Canada; 2 Department of Veterinary Microbiology, Saskatoon, SK Canada; 3 Prairie Diagnostic Services Inc, Saskatoon, SK Canada; 4 Department of Farm Animal Health, Faculty of Veterinary Medicine, Utrecht University, Utrecht, The Netherlands; 5 Western College of Veterinary Medicine, University of Saskatchewan, Saskatoon, SK S7N 5B4 Canada

**Keywords:** Mouse, CF-1, C3H, TD85420, Inoculation, Swine dysentery, “*Brachyspira hampsonii”*, *Brachyspira hyodysenteriae*, Typhlocolitis

## Abstract

**Background:**

The development of a mouse model as an in vivo pathogenicity screening tool for *Brachyspira* spp. has advanced the study of these economically important pathogens in recent years. However, none of the murine models published to date have been used to characterize the clinical signs of disease in mice, instead focusing on pathology following oral inoculation with various *Brachyspira* spp. The experiments described herein explore modifications of published models to characterize faecal consistency, faecal shedding and pathology in mice challenged with “*Brachyspira hampsonii”* clade II (Bhamp).

**Methods and results:**

In Experiment 1, 24 CF-1 mice were randomly allocated to one of three inoculation groups: sham (Ctrl), Bhamp, or *B. hyodysenteriae* (Bhyo; positive control). Half of each group was fed normal mouse chow (RMH) while the other received a low-zinc diet (TD85420). In Experiment 2, eight CF-1 mice and nine C3H/HeN mice were divided into Ctrl or Bhamp inoculation groups, and all fed TD85420. In Experiment 1, mice fed TD85420 demonstrated more severe mucoid faeces (*P* = 0.001; Kruskal Wallis) and faecal shedding for a significantly greater number of days (*P* = 0.005; Kruskal Wallis). Mean faecal scores of Bhamp inoculated mice trended higher than Ctrl (*P* = 0.06; Wilcoxon rank-sum) as did those of Bhyo mice (*P* = 0.0; Wilcoxon rank-sum). In Experiment 2, mean faecal scores of inoculated CF-1 mice were significantly greater than in C3H mice (*P* = 0.049; Kruskal Wallis) but no group differences in faecal shedding were observed. In both experiments, mice clustered based on the severity of colonic and caecal histopathology but high lesion scores were not always concurrent with high fecal scores.

**Conclusion:**

In our laboratory, CF-1 mice and the lower-zinc TD85420 diet provide a superior murine challenge model of “*Brachyspira hampsonii”* clade II.

**Electronic supplementary material:**

The online version of this article (doi:10.1186/s12917-017-1166-5) contains supplementary material, which is available to authorized users.

## Background

In 2009, a new species of *Brachyspira* associated with disease resembling SD was discovered on a Western Canadian pig farm [1] and was subsequently shown to experimentally reproduce mucohaemorrhagic diarrhoea and typhlocolitis in orally inoculated pigs [2, 3]. Initially called *Brachyspira* sp. Sask30446, the species has now been provisionally named as “*Brachyspira hampsonii*” [4, 5]. Strains 30,599 and 30,446 are Canadian reference strains of clade I and II, respectively. The discovery of a new, pathogenic species affecting the swine industry necessitates continued study with a focus on better understanding disease pathogenesis and control including vaccine development.

Several *Brachyspira* spp. that are pathogenic in pigs, are known to exploit rodents and waterfowl species as natural reservoirs [6–9]. The ability of *Brachyspira* spp. to colonize rodents has led to the development of mouse models of SD. The first published model used CF-1 mice fed a standard rodent chow diet to determine whether it was possible to experimentally infect mice with *Treponema hyodysenteriae* [10, 11]. Not only did the mice in this study become infected, they also developed caecal lesions typical of SD in pigs. The model was subsequently modified by using various strains of C3H mice and a defined diet (TD85420; Harlan Laboratories, Indianapolis, IN) to obtain more consistent caecal lesions following oral inoculation with various isolates of *Brachyspira* spp. [12–14]. Because mice have a small caecum, a swine dysentery challenge model using 24 h chicks was developed in the mid-1980s [15, 16], and has since been used to study a number of pathogenic and non-pathogenic *Brachyspira* spp. [17, 18]. Chicks have two large caeca and develop mild to moderate typhlitis following *B. hyodysenteriae* infection [15], however, subtle changes in faecal consistency are less easily judged due to the nature of their droppings. In spite of being a relevant, non-invasive indicator of virulence and disease severity, changes in faecal consistency have not yet been documented in infection models using chicks or mice, including the most recent and only mouse challenge assessing the pathogenicity of “*B. hampsonii”* [19].

The overarching objective of the two pilot experiments reported herein was to optimize a pre-existing *Brachyspira* mouse model of swine dysentery for *“B. hampsonii*” clade II (strain 30,446), with emphasis on the assessment of clinical signs of disease following oral challenge to ensure it is suited for use evaluating oral and parenteral vaccine prototypes. The primary objectives of Experiment 1 were to determine if CF-1 mice are susceptible to infection with *“B. hampsonii”* strain 30,446, and to determine if the low zinc TD85420 diet affected the severity of clinical disease. A *B. hyodysenteriae* infected group was included as positive control, and enabled a secondary objective of comparing disease severity between two *Brachyspira* species, albeit with low experimental power. The primary objective of Experiment 2 was to compare disease severity in CF-1 and C3H mice following *“B. hampsonii”* challenge to determine if there are differences in susceptibility between the two mouse strains.

## Methods

### Experiment 1

#### Mice

Twenty-four, 4-week old, specific pathogen free, female CF-1 mice (Charles River Laboratories, Kingston, NY were obtained from a commercial supplier and randomly assigned to one of three inoculation groups: sham (Ctrl; *n* = 8), “*Brachyspira hampsonii”* clade II strain 30,446 (Bhamp; *n* = 8), or positive control *B. hyodysenteriae* (Bhyo; *n* = 8). Each inoculation group was divided into two plastic cages (6 cages total, *n* = 4 per cage). One cage per group was fed normal rodent chow, RMH 3000 (RMH; Federated Co-Operatives Limited, Saskatoon, SK, Canada), and the other was fed a defined low zinc, Teklad diet TD85420 (TD) for the duration of the experiment. Each mouse was assigned a unique identity by tail-mark and was allowed to acclimate for 7 days prior to inoculation. Mice were housed in ventilated, biocontainment level 2 cage racks with aspen shavings, which were changed once per week. Mouse houses and crinkle bedding were provided in each cage for enrichment.

#### Bacterial strains and culture

The “*B. hampsonii*” strain 30,446 and *B. hyodysenteriae* strain G44 (generously provided by Boehringer Ingelheim Vet Medica, St Joseph, MO) used for inoculation were cultured in JBS broth (BHI plus 0.5% (*v*/v) sheep blood and 0.5% (*v*/v) glucose) [2]. To detect *Brachyspira* in samples from mice, faecal samples and terminal caecal content swabs were cultured on BJ agar [20] at 42 °C using an anaerobic gas pack (Oxoid Anaerogen™, Thermo Fisher Scientific, Waltham, MA) for a total of 4 days, with readings taken at 48 and 96 h post-plating. Appearance of zones of strong β-haemolysis on selective BJ agar was considered a presumptive positive culture result.

#### Molecular identification and quantification of *Brachyspira*

Total DNA was purified from 1 mL samples of broth cultures using a commercial kit (DNeasy Blood and Tissue Kit, Qiagen, Mississauga, ON) and the concentration of “*B. hampsonii”* 30,446 or *B. hyodysenteriae* in inoculum preparations was determined using clade- or species-specific SYBR green quantitative real-time PCR with primers as previously described [2]. Results were expressed as logarithm base 10 genome equivalents (GE) per mL.

For confirmation of species identity from culture-positive faecal or terminal caecal samples, zones of strong β-haemolysis were sampled with a sterile toothpick and subjected to genus-specific *Brachyspira* PCR using previously published primers targeting the NADH oxidase (*nox*) gene [21]. PCR products were purified and sequenced using the amplification primers. Raw sequence data was assembled and edited using Pregap4 and Gap4 [22] and the resulting sequences compared to published *nox* sequences for identification.

#### Inoculation procedure

The mice underwent a 7-day acclimatization period, during which they were fed exclusively the appropriate diet for their experimental group. Water and food were provided ad libitum throughout the experiment. For each of two daily inoculations (2:00 p.m.), mice were restrained using the double handed manual technique, then held vertically by the scruff and inoculated intra-gastrically using a straight, 20 gauge blunt-ended feeding needle. The inocula was 0.2 mL of a 24 h old broth culture of the relevant *Brachyspira* species on day 0 (Bhyo = 1.6 × 10^8^, Bhamp = 1.2 × 10^9^ GE per 200 μL dose) and one (Bhyo = 1.0 × 10^8^, Bhamp = 1.56 × 10^9^ GE per 200 μL dose) day post-inoculation (dpi). The negative control (Ctrl) mice were sham inoculated with sterile broth in the same manner. The mice were then allowed to recover in their original cages. No sedation was used.

#### Clinical assessments

Mice were monitored by research personnel twice daily (8:30 a.m. and 3:00 p.m.) to assess signs of illness based on physical appearance and faecal consistency. At each observation, mice were held by the tail and placed on the examiner’s palm to facilitate simultaneous defecation. Faeces were observed for the presence of mucus and/or blood, and graded using a scoring system modified from previous pig challenge experiments conducted in our laboratory [2, 23](Additional file 1): Score 0 = formed faecal pellet, 1 = formed pellet with a trace mucous tail, 2 = soft, mucoid faeces, 3 = faeces with blood (+/− mucus). From each mouse, faecal pellets were collected at −2 and 0 dpi for culture and *nox* PCR to confirm that mice were *Brachyspira*-free prior to inoculation. Faecal samples were also collected from individual mice after inoculation at 5, 7, 9, and 12 dpi to monitor *Brachyspira* shedding.

#### Pathological assessments

The experiment was terminated at 15 dpi. At this time, mice were euthanized by way of anaesthetic overdose (isoflurane gas inhalation; AErrane™ Baxter Corporation, Mississauga, ON, Canada) followed by exsanguination. A necropsy was performed immediately thereafter with focus on the gastrointestinal tract. Fresh samples of caecum and colon were collected for culture and *nox* PCR. The carcass was placed in 10% neutral buffered formalin for 24 h. and samples of ileum, colon, caecum, heart, lung, salivary gland, rectum, liver, spleen, kidney and stomach were processed routinely for histological analysis of 5 μm haematoxylin and eosin stained (H and E) sections by a board certified veterinary pathologist blinded to treatment group. For Experiment 1, the presence or absence of catarrhal inflammation and epithelial regeneration in the colon and caecum were recorded.

#### Fluorescent in situ hybridization (FISH)

To better visualize the interaction of *Brachyspira* organisms on the colonic mucosae and crypts, 2 cases from each *Brachyspira* group, selected on the basis of high fecal consistency score, lesion severity and *Brachyspira* DNA concentration in colon, along with 1 control case were selected for FISH targeting a *Brachyspira* generic probe. The staining protocol was adapted from previous work [24, 25]. Paraffin embedded colonic tissue sections were cut at 4 μm and mounted on histology slide (FisherBrand Superfrost *plus*, Thermo Fisher Scientific Inc., MA., USA). Tissue sections were deparaffinized by baking in a rotary oven (BK200 Roto-Dry Slide Dryer, Mopec, MI, USA) for one hour at 65 °C, followed by three 10 min. Xylene serial passages. Tissues were dehydrated by passage through 100% ethanol (2 passages, 10 min. Each), 95% ethanol (5 min.), and 70% ethanol (5 min.), and allowed to air dry at room temperature. A previously described [24], a custom fluorophore-labelled DNA probe, SER1410 (Alexa 546–5′-GTCATTCCATCGAAACATA- 3′) designed for *Brachyspira* spp. was obtained from a commercial source (Invitrogen Corporation, CA, USA) and reconstituted using nuclease free-water to a working concentration of 5 ng/μl in hybridization buffer (20 mM Tris [pH 7.2], 0.9 M NaCl, 0.1% sodium dodecyl sulfate [*w*/*v*],40% formamide [*w*/*v*], 10% dextran sulfate). Tissue sections were covered with 200 μl of hybridization solution, placed in a hybridization oven (Boekel Scientific, Feaesterville, PA, USA), and hybridized overnight at 45 °C. Following hybridization, tissue sections were washed in wash buffer (20 mM Tris [pH 7.2], 0.9 M NaCl, 10% dextran sulfate [*w*/*v*]) pre-warmed to 45 °C for 20 min., rinsed with sterile water, and air dried. Slides were mounted on antifading agent (ProLong® Gold Antifade Reagent, Life Technologies, Carsbad, CA, USA) and covered with 0.13–0.17 mm thickness coverglass (Fisherbrand #1.5 cover glass, Thermo Scientific, MI, USA). Mounted slides were kept at 4 °C until analyzed.

The slides were visualized using a wide-band epifluorescence microscope (Olympus IX83, Olympus Corporation, Tokyo, Japan) under 100× objective lens (Olympus UPlanSApo 100×, 1.40, oil, Olympus Corporation, Tokyo, Japan). Filters corresponding to GFP/Green (LED excitation - 460 nm, emission - ET525/50) and RFP/Red (LED excitation - 565 nm; emission – ET630/75) were used for multicolour imaging. Images were captured using Andor Zyla sCOMS camera (Andor Technologies, Belfast, UK) and an imaging software (Olympus cellSens imaging software, Olympus Corporation, Richmond Hill, ON, Canada).

### Experiment 2

Procedures for Experiment 2 were similar to above with a few exceptions described below.

#### Mice

Four-week old, specific pathogen free, female CF-1 (*n* = 8) and C3H/HeN (“C3H”, *n* = 9) mice were randomly assigned to one of two inoculation groups as follows: CF-1-Ctrl (*n* = 4), C3H-Ctrl (*n* = 4), CF-1-Bhamp (*n* = 4), and C3H-Bhamp (*n* = 5). All mice were fed TD85420 diet and allowed to acclimate for 14 days prior to inoculation.

#### Inoculation procedure

Because the mice were 1 week older at inoculation than those used in Experiment 1, the inoculum dose was increased to 0.3 mL per mouse of *“B. hampsonii*” broth culture containing 2.31 × 10^8^, 2.22 × 10^8^, 1.33 × 10^8^ GE per 300 μL dose, on D0, D1, D2, respectively. The mice also underwent a 6-h fasting period prior to each of three intra-gastric inoculations to decrease gastric transit time. Inoculation procedures were identical to Experiment 1.

#### Molecular identification and quantification of *Brachyspira*

To confirm the detection of *“B. hampsonii*” 30,446 in pre-mortem faecal samples and terminal caecal samples, zones of strong β-haemolysis were sampled with sterile toothpicks and subjected to clade-specific conventional PCR using the same primers employed in the quantitative PCR assay used in Experiment 1 [2]. PCR reactions contained 2.5 U *Taq* DNA polymerase (Quanta Bio Sciences, MD, USA), 2.5 mM MgCl_2,_ 50 mM KCl, 10 mM Tris/HCl pH 8.3, 250 μM of each dNTP and 20 pmol each of primers JH0224 and JH0225. PCR reactions were incubated at 94 °C for 3 min. Followed by 40 cycles of (15 s. at 94 °C, 15 s. at 60 °C and 30 s. at 72 °C) and a final extension of 5 min at 72 °C. No template controls were run with each set of PCR reactions. A visible band of 215 bp on a 1% agarose gel confirmed detection of “*Brachyspira hampsonii*” stain 30,446.

#### Clinical assessments

Clinical assessments were performed twice daily as previously described for Experiment 1. The faecal scoring system was refined to distinguish “mild” from “severe” soft, mucoid faeces. Slightly soft and mucoid faeces were scored as 2.0, whereas very soft and mucoid faeces were scored 2.5 (Additional file 1). Faecal samples were collected from each mouse at −2 and 0 dpi for culture and *nox* PCR to confirm that the mice were *Brachyspira*-free prior to inoculation.

#### Pathological assessments

The experiment was terminated at 14 dpi. The entire colon was linearized and five sections throughout its length were preserved in Carnoy’s solution, instead of 10% neutral buffered formalin, to allow for better preservation of the mucous layer and structural integrity of intestinal tissue [26]. Alternating sections were collected for histopathology, *Brachyspira* culture and PCR. The depth of crypts was assessed in H and E stained sections of colon by measuring the distance between the bottom and top of intact crypts using an eyepiece reticle. Measurements were performed by an observer blinded to treatment group on the available colonic sections (range 9–16) for each mouse, with 5 measurements per section. The following histological changes in the colon and caecum were semi-quantitatively graded from 0 to 3 (Additional files 2, 3, 4 and 5): catarrhal inflammation, epithelial regeneration, degree of mucus accumulation and neutrophilic infiltration.

### Statistical analyses

All statistical analyses were performed using Stata v14 (StataCorp, College Station, TX). Group differences were deemed statistically different if *P* < 0.05, and a trend towards significance if 0.05 ≤ *P* < 0.1 a priori*.*

### Experiment 1

A mean faecal score per mouse was calculated by averaging the twice daily scores from 3 dpi to termination. Fecal consistency scores from 0 to 2 dpi were not considered in this calculation because the intra-gastric infusion of liquid media (200–300 μl) periodically results in a loose pellets. Mean faecal scores and the number of days that the inoculated *Brachyspira* (Bhyo or Bhamp) was cultured from faeces (total shedding days) were compared between diet and *Brachyspira* spp. using a Kruskal Wallis test and post-hoc Wilcoxon rank-sum test where needed. The incubation period was defined as the number of days required for a mouse to develop consistent soft mucoid faeces (score 2) during the post-inoculation period. Mice that did not develop soft mucoid faeces were assigned a value of 15 d (i.e. censored at termination). The incubation period was compared between *Brachyspira* spp. and diet using a Kaplan Meier survival curve followed by Wilcoxon and log-rank tests for equality of survivor functions. Finally, the relationship of histopathologic lesions (catarrhal inflammation, epithelial regeneration) in caecum and colon to *Brachyspira* group, diet and clinical outcome was assessed using cluster analysis (Ward’s linkage; Matched Similarity Measure). The appropriate number of clusters were determined using the post-hoc Calinski-Harabasz pseudo-F index and Duda-Hart Je (2)/Je (1) index stopping rules.

### Experiment 2

Similar analyses was undertaken to determine potential differences between mouse strains following *“B. hampsonii”* challenge. In addition, separate multilevel, linear regression models (XTMIXED) were used to investigate differences in crypt depth between mouse strains and inoculation status. For these, mouse identification was included as a random intercept. The relationship of histopathologic lesions (catarrhal inflammation, neutrophil infiltration, mucus accumulation, mucus epithelial regeneration) in caecum and colon to *Brachyspira* spp., mouse strain and clinical outcome was assessed using cluster analysis (Ward’s linkage; Euclidean Dissimilarity Measure). Because the histopathology data was ordinal, cluster analysis was performed on scaled-ranks as described by Kaufman and Rousseeuw [27].

## Results

All pre-inoculation faecal samples were culture-negative for *Brachyspira*, indicating no *Brachyspira* infection was present prior to experimental inoculation, or if present, was below the detection limit of selective culture.

### Experiment 1

#### Clinical signs

Both Bhamp and Bhyo inoculation groups exhibited mucoid faeces following inoculation (Additional file 6). From 3 dpi onwards, mean faecal scores across both dietary groups in Bhyo were significantly higher than in sham-inoculated Ctrl (*P* = 0.007; Kruskal Wallis, post hoc Wilcoxon rank-sum), whereas Bhamp inoculated mice similarly trended (*P* = 0.06; Wilcoxon rank-sum). Increased mean faecal scores were particularly apparent in mice fed TD85420. Soft, mucoid faeces were observed after inoculation in 100% of mice from the Bhamp and Bhyo groups fed TD85420, compared to only half of the Bhyo and none of the Bhamp mice fed RMH chow. Overall, mean faecal consistency scores were higher in the TD85420 compared to the RMH group, and this result was consistent in both *Brachyspira* groups as well as Ctrl (*P* = 0.02 for Bhyo and Bhamp; *P* = 0.04 for Ctrl; Kruskal Wallis with post-hoc Wilcoxon rank-sum). The severity of clinical disease, however, did not differ between Bhyo and Bhamp groups (Table 1).

**Table 1:** Summarized pathologic lesion results from Experiment 1 evaluating *Brachyspira* spp. and diet^a^ in CF-1 mice

				Colon		Caecum	
Species Group	Diet	Presence of soft mucoid faeces	Mean faecal consistency score^b^	Catarrhal inflammation	Epithelial regeneration	Catarrhal inflammation	Epithelial regeneration
Ctrl	RMH	0/4	0.034	1/4	0/4	0/4	0/4
Ctrl	TD	0/4	0.210	0/4	0/4	0/4	0/4
Bhamp	RMH	0/4	0.161	0/4	0/4	0/4	0/4
Bhamp	TD	4/4	0.823	1/4	0/4	1/4	2/4
Bhyo	RMH	2/4	0.272	2/4	0/4	0/4	0/4
Bhyo	TD	4/4	0.867	2/4	0/4	0/4	2/4

Legend: *Ctrl* non-inoculated group, *Bhamp “B. hampsonii”* inoculated group, *Bhyo B. hyodysenteriae* inoculated group, *RMH* RMH3000 chow diet (normal zinc), *TD* TD85420 diet. See Additional files 1, 2, 3, 4 and 5 for description of histopathologic lesion scores

^a^Values indicate the numbers (proportions) of mice with each type of clinical sign or lesion

^b^Mean fecal consistency score calculated by averaging twice daily observations from 3 dpi to termination. Mean fecal score differs across species: Bhyo vs Ctrl (*P* = 0.007); Bhamp vs Ctrl (*P* = 0.06). Mean fecal score differs by diet (TD vs RMH): *P* = 0.02 for Bhyo and Bhamp, *P* = 0.04 for Ctrl

Faecal shedding (total shedding days) was also elevated in mice fed TD85420 (*P* = 0.005; Kruskal Wallis), but did not differ between the Bhyo and Bhamp infected groups. All culture-positive plates from terminal caecum samples were confirmed to be the inoculated strain via *nox* PCR and sequencing.

The incubation period was significantly shorter in TD85420 fed mice (*P* = 0.01; Kaplan Meier with post-hoc log-rank and Breslow tests) (Additional file 7a), which resulted from more rapid onset and more prevalent soft, mucoid faeces.

#### Pathology

**Fig. 1:**
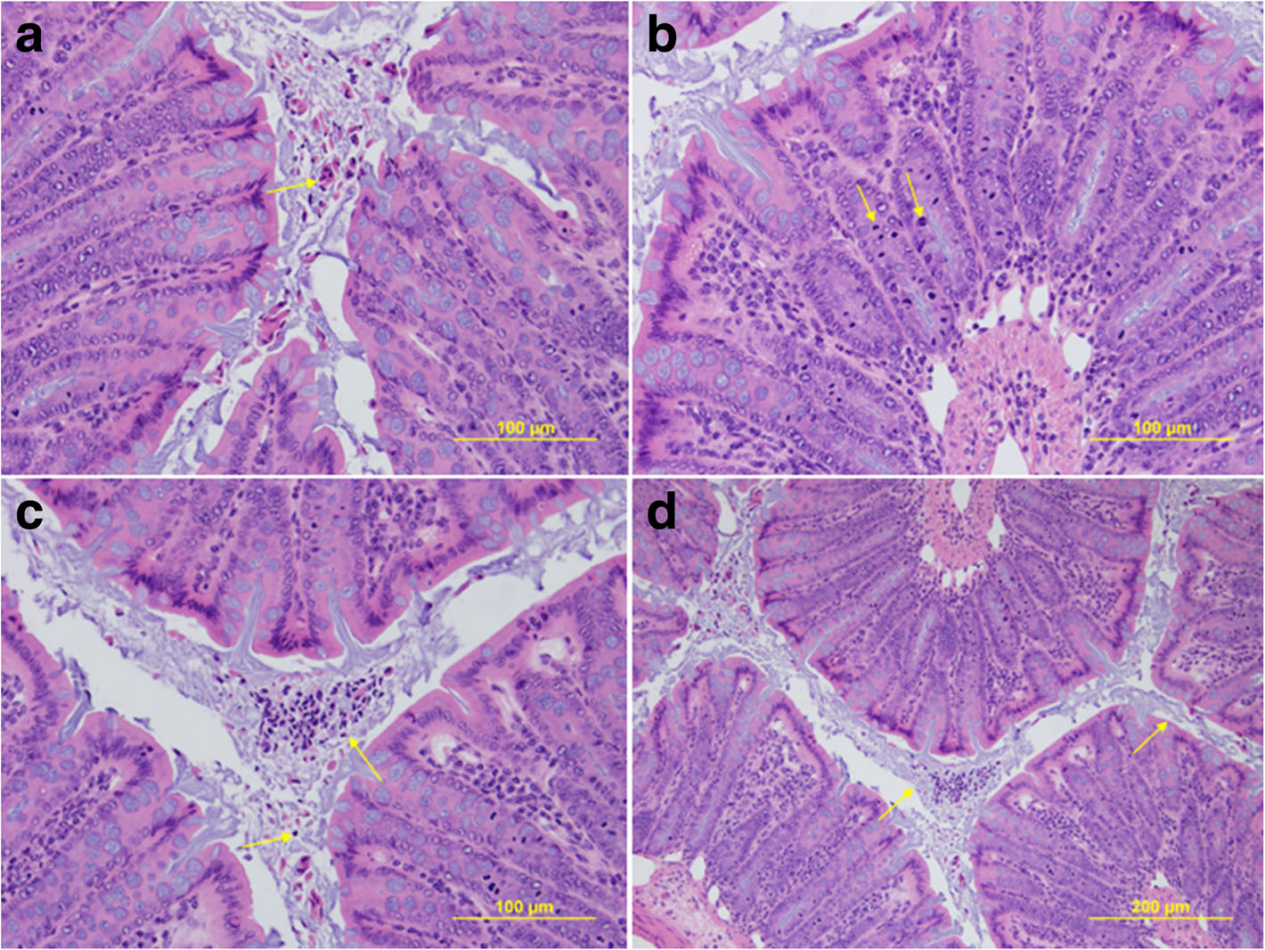
Representative histopathologic lesions in Experiment 1 and 2 mice. **a** catarrhal inflammation showing groups of degenerative epithelial cells (*arrow*) in the lumen of colon; 40×. **b** epithelial regeneration showing very frequent mitotic figures (*arrows*) are noted in the hyperplastic colonic crypts; 40×. **c** neutrophilic infiltration, showing neutrophils (*arrow*) in the lumen and in the lamina propria of the colon; 40×. **d** mucoid exudate showing large lakes of basophilic mucus (*arrow*) in the lumen of colon; 20 ×

**Fig. 2:**
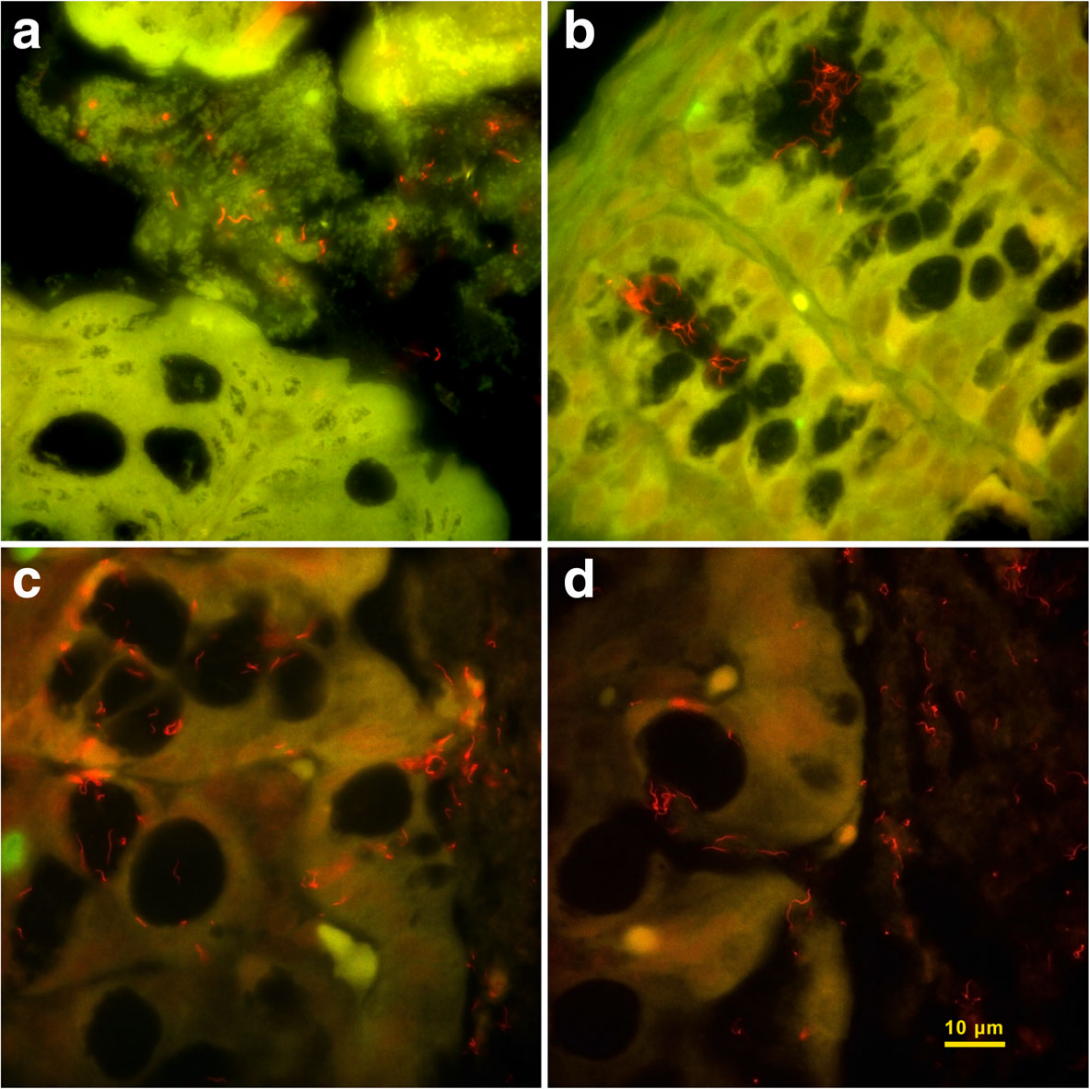
Representative fluorescence in situ hybridization images of colon from Experiment 1 mice. *Brachyspira* cells are observed with strong positive signal (*red serpentine-shaped cells*), whereas non-specific cellular fluorescence appears green/yellow. **a** Large numbers of spirochaetes are observed within the luminal mucus of “*B. hampsonii”* infected mouse (#2) with no lesions fed RMH diet; **b** Large numbers of spirochaetes are observed within the colonic crypts of a *B. hyodysenteriae* infected mouse (#19) fed RMH diet with catarrhal inflammation in colon. **c** & **d**) Spirochaetes in close proximity with colonic epithelium and goblet cells from a *B. hyodysenteriae* infected mouse (#16) fed TD85420 diet with catarrhal inflammation in colon

**Fig. 3:**
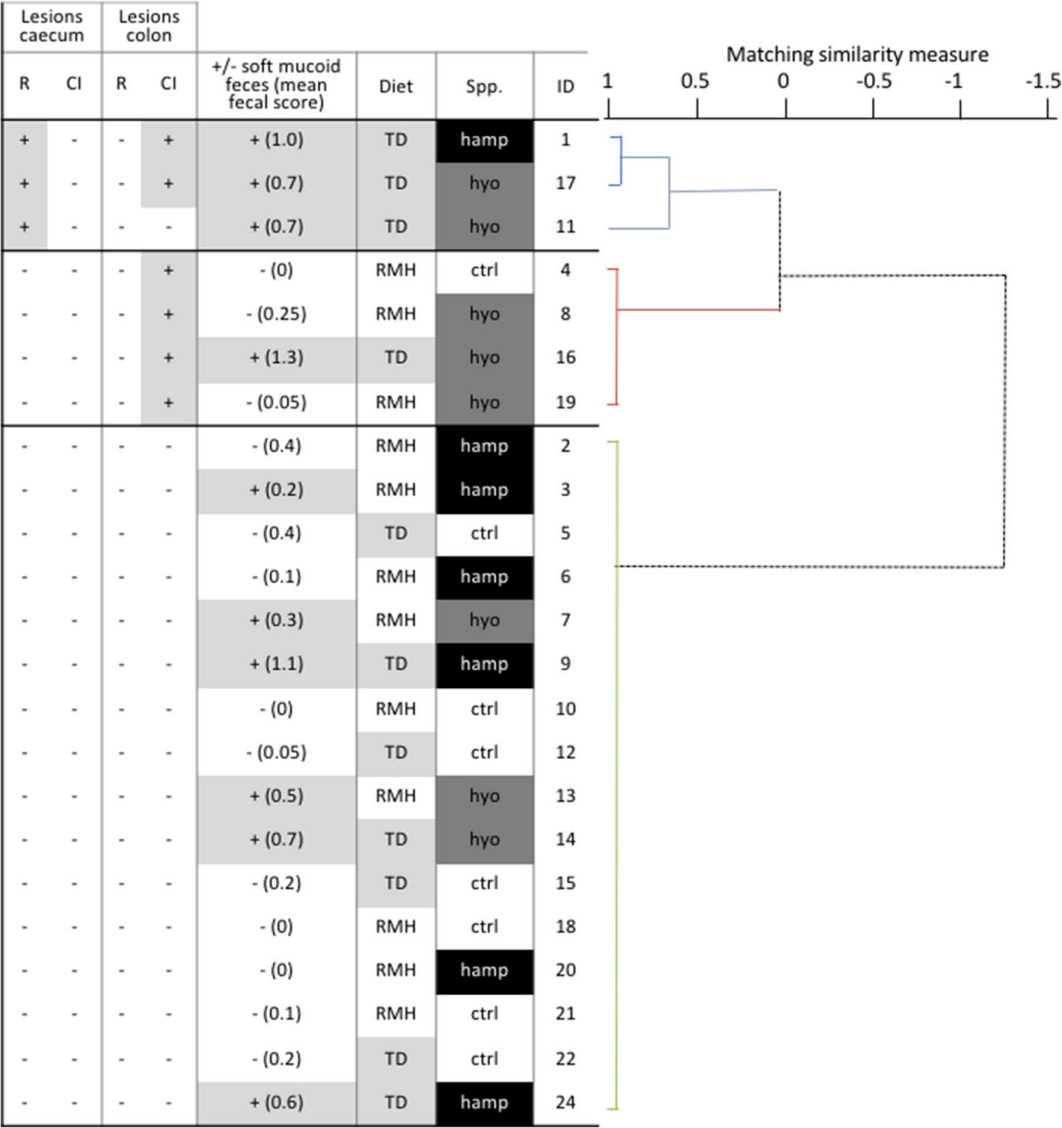
Cluster dendrogram and data table for Experiment 1 showing clustering of histopathology. Three clusters (*Ward’s linkage, Matched Similarity Measure*) are evident; all unrelated to *Brachyspira* species, diet or clinical outcome. Cluster 1 (*blue*) distinguishes mice with epithelial regeneration in caecum. Two of three mice also have a catarrhal inflammation in colon, as do all mice in cluster 2 (*red*). Cluster 3 (*green*) includes mice with no pathology in caecum and colon. R = regeneration of epithelium; CI = presence of catarrhal inflammation; TD = low zinc TD85420 diet; RMH = RMH3000 chow diet (normal zinc); spp. = *Brachyspira* species used for inoculation; hamp = “*B. hampsonii*”; hyo = *B. hyodysenteriae*; ctrl = non-inoculated control; ID = mouse identification; + = lesion or soft mucoid faeces present; − = lesion or soft mucoid faeces absent

The most prevalent lesion in infected mice was catarrhal inflammation of the colon, characterized by sloughing of superficial epithelial cells, which were frequently degenerated and mixed with mucus (Fig. 1a). Still, only 25% of Bhamp-TD and 50% of Bhyo-TD mice had catarrhal lesions (Table 1). Epithelial regeneration, characterized by crypt hyperplasia, increased cytoplasmic basophilia, and increased mitotic activity (Fig. 1b) was less commonly observed, and only noted in 50% of Bhamp-TD mice (Table 1). *Brachyspira* organisms were seen in FISH-stained colonic tissue of mice inoculated with Bhyo and Bhamp, whereas samples from control mice did not reveal any positive signals. In infected mice, *Brachyspira* spirochaetes were observed in the luminal mucus as well as in crypts in close proximity to epithelial and goblet cells (Fig. 2). Mice clustered into three discrete clusters based on the presence or absence of histopathological lesions present in their colon and caecum (Fig. 3). The largest cluster (#3) included mice with no lesions. With one exception, clusters 1 and 2 were based on the presence of lesions in colon only (#2) versus colon and caecum (#1). Unexpectedly, the clustering of histopathology was unrelated to *Brachyspira* species, diet and clinical outcome.

No mouse demonstrated any evidence of other systemic or intestinal diseases, based on gross and histopathologic examination.

### Experiment 2

#### Clinical signs

CF-1 mice exhibited more severe clinical disease, with 3 of 4 Bhamp inoculated mice developing score 2.0 faeces compared to only 1 of 5 inoculated C3H mice (Additional file 8). The mean faecal scores of CF-1 mice were significantly greater than C3H mice (*P* = 0.049; Kruskal Wallis; Table 2). Although faecal *Brachyspira* shedding did not differ by mouse strain, there was a trend toward a shorter incubation time in CF-1 versus C3H mice (*P* = 0.08 Kaplan Meier with post-hoc log-rank and Breslow tests) (Additional file 7b).

**Table 2:** Summarized pathologic lesion results from Experiment 2 evaluating mouse strain^a^

				Colon			Caecum		
Mouse strain	Species Group	Presence of soft mucoid faeces	Mean faecal consistency score^b^	Catarrhal inflammation	Neutrophilic infiltration	Epithelial regeneration	Catarrhal inflammation	Neutrophilic infiltration	Epithelial regeneration
CF-1	Ctrl	0/4	0.031	2/4	0/4	0/4	0/4	0/4	0/4
C3H	Ctrl	0/4	0.000	2/4	0/4	0/4	0/4	0/4	0/4
CF-1	Bhamp	3/4	0.988	3/4	2/4	3/4	1/4	0/4	3/4
C3H	Bhamp	1/5	0.113	2/5	2/5	2/5	4/5	2/5	2/5

Legend: *Ctrl* non-inoculated group, *Bhamp “B. hampsonii”* inoculated group, CF*-1* CF-1 mouse strain, *C3H* C3H mouse strain. See Additional files 1, 2, 3, 4 and 5 for description of histopathologic lesion scores

^a^Values indicate the numbers (proportions) of mice with each type of clinical sign or lesion

^b^Mean fecal consistency score calculated by averaging twice daily observations from 3 dpi to termination. Mean fecal score differs across mouse strain: CF-1 vs C3H (*P* = 0.049)

#### Pathology

**Fig. 4:**
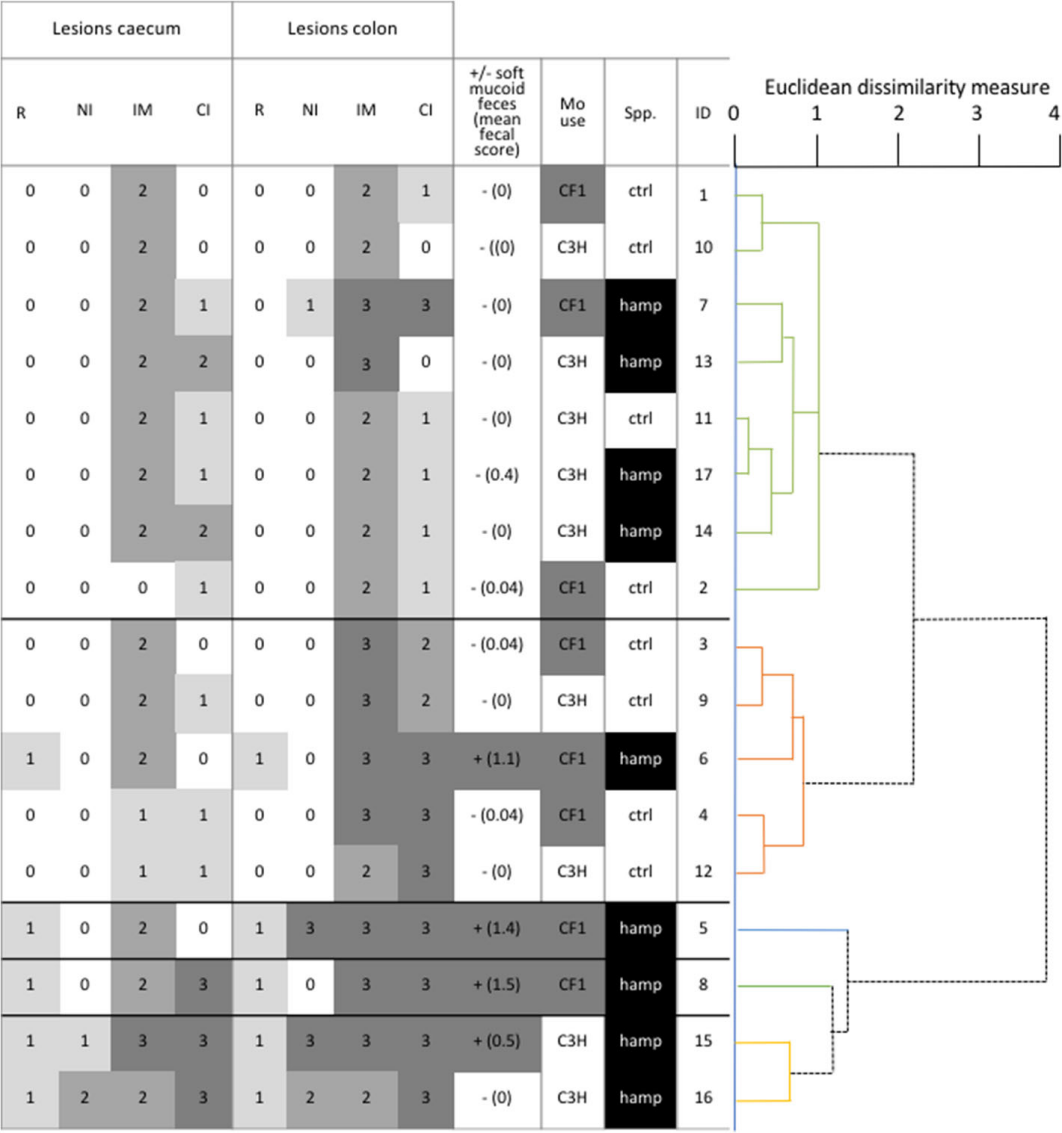
Cluster dendrogram and data table for Experiment 2 showing clustering of histopathology. Five clusters (Ward’s linkage of rank-scaled scores, Euclidean dissimilarity measure) are evident; unrelated to mouse strain, but associated with clinical outcome. Values in lesion columns represent the lesion severity score of colonic and caecal tissue sections, with more intense grey scale indicating mice with more prevalent lesions. The single mice in cluster 3 (*blue*) and 4 (*orange*) had the highest mean faecal scores and most severe colonic and caecal lesions. One inoculated mouse in cluster 2 (*red*) also had severe intestinal pathology and high faecal score. Cluster 5 (*purple*) included mice with severe pathology but both had low mean faecal scores. Mice with the least severe clinical disease and pathology were grouped in cluster 1 (*green*). Clusters R = regeneration of epithelium; NI = neutrophilic inflammation; IM = increased mucoid exudate; CI = catarrhal inflammation; CF1 = CF-1 mouse strain; C3H = C3H mouse strain; Spp. = *Brachyspira* species used for inoculation; hamp = “*B. hampsonii*”; ctrl = non-inoculated control; ID = mouse identification; + = soft mucoid faeces present; − = soft mucoid faeces absent

Similar histological changes described in Experiment 1 were noted (Table 2). In addition, neutrophilic infiltration in the lamina propria and submucosa were evident in small numbers of mice (Fig. 1c). The mice formed discrete clusters based on the severity of histopathological lesions (Fig. 4). In this experiment, five clusters of varying size were evident. Increased mucous exudate in colon and caecum (Fig. 1d) was observed similarly across all clusters. Cluster 1 and 2 included mice observed with a mild and moderate catarrhal inflammation in caecum and colon. Cluster 3 to 5 featured only Bhamp-inoculated mice with evidence of more severe lesions, including catarrhal and neutrophilic inflammation, and epithelial regeneration. Cluster 3 and 4 comprised of only one mouse each, but had the most severe clinical signs and lesions, compared to all other clusters.

Across all mice, crypt depth averaged 161 ± 49 μm. There was a trend towards C3H mice having lower crypt depth than CF-1 (*P* = 0.08; XTMIXED), but the C3H mice were 3.5 g lighter than CF-1 at termination which may explain this result. Crypt depth did not differ between inoculated and control mice at termination.

Based on gross and histopathological examination, none of the mice demonstrated any evidence of other systemic or intestinal diseases.

## Discussion

The two mouse inoculation experiments outlined here indicate that it is possible to consistently induce soft, mucoid faeces following experimental challenge with “*Brachyspira hampsonii*” clade II in mice under ideal conditions. From the results of Experiment 1, it is clear that TD85420 enhances *Brachyspira*-associated disease severity in mice. This finding is consistent with previous pathological assessments in which caecal lesions were found to be most severe in mice fed TD85420 [13, 19]. The mechanism by which this is achieved is not fully understood, but could be attributed to the low zinc levels of TD85420. Zinc supplementation was previously shown to have a prophylactic effect against *B. hyodysenteriae* challenge in mice [28], which may be associated with reduced biosynthesis of haemolysin toxin rather than inhibition of pre-formed haemolysin [29]. Zinc supplementation may also decrease *Brachyspira* colonization in the gut as zinc (supplied as zinc oxide, ZnO) is commonly used as a supplement in swine production diets to promote weight gain [30] and maintain stability of intestinal microbiota post-weaning [31, 32]. The TD85420 diet also includes an elevated fat content, which may contribute to its effects [33].

The CF-1 mouse strain was more severely affected than C3H mice in terms of faecal consistency. However, faecal shedding of *Brachyspira* spp. was not different between CF-1 and C3H mice. This indicates that although both mouse strains became colonized by *“B. hampsonii”* clade II to similar extents, this level of infection resulted in more severe disease in CF-1 mice. The reason for this apparent difference is difficult to ascertain without further study, but may be attributed to genetics. The inbred C3H strains were first selected for this type of experiment because of their varying degrees of responsiveness (determined by the *lps* locus) to lipopolysaccharide (LPS), a component of the outer membrane of Gram-negative bacteria. Although it was proposed that LPS hypo-responsive mouse strains would be less susceptible to lesion development following *Serpula hyodysenteriae* challenge than those mouse strains with greater responsiveness, the study found that other inherent properties are also likely to be involved in determining susceptibility to *S. hyodysenteriae* infection and expression of disease [14].

Assessing faecal consistency was a very valuable measure of clinical disease in these experiments, with inoculated mice exhibiting more frequent soft, mucoid faeces than controls. The scoring system developed herein to assess faecal consistency in mice was modified from those used in pig inoculation studies [2, 23]. During Experiment 1, it became clear that our initial faecal consistency categories were too simplistic to cover the wide range in soft mucoid faeces observed during the experiment. For this reason, the faecal scoring system was modified for Experiment 2 by adding scoring category of 2.5 to record faeces that were very soft and mucoid (Additional file 1). Twice daily assessment of faecal consistency was important because morning and afternoon faecal scores often differed, and mice occasionally did not defecate during one of the daily faecal scoring periods. Faecal consistency assessments are non-invasive, require minimal staff training to implement, and provide valuable, relevant and inexpensive clinical data. The data collected in these experiments helps to demonstrate that mice are capable of developing clinical enteric disease and emphasizes the suitability of mice for *Brachyspira* studies.

Faecal shedding followed a similar pattern to faecal consistency in Experiment 1, but may be influenced by differences in faecal consistency between mice. Softer faecal samples were much easier to plate for culture compared to solid faecal pellets and thus the amount of faeces deposited on each plate was difficult to control. This added a source of variation in the culture procedure used to assess shedding. Measuring *Brachyspira* DNA concentration in weighed samples by quantitative PCR would partially overcome this issue, although the results could still be influenced by faecal moisture content to some extent. Measuring faecal content in mouse droppings should also be considered.

The two mouse strains differed greatly in weight at arrival and this may have potentially biased the evaluation of assessment of intestinal crypt depth in Experiment 2. Although crypt depth measurement has been used in pig challenge studies as an indicator of disease [34, 35] it appears to have limited utility in mice.

Unexpectedly, the pathologic results in these experiments were not completely consistent with what has been reported in previous studies of *Brachyspira* challenged mice [13, 14, 19, 36]. The catarrhal changes noted in the current experiments were not reported previously, and the neutrophilic infiltration reported in previous studies was only evident in Experiment 2. The reasons for the discrepancies are not known. It may be that the previous studies mostly focused on the cellular infiltrates in the lamina propria, thus the catarrhal changes were not recorded. The lack of neutrophilic infiltration in Experiment 1 may be the effect of differences in challenge dose and protocol. This may be supported by the fact that, in Experiment 2, where inoculation dose was larger, there were higher rates of neutrophilic infiltration. Mouse origin can also affect susceptibility to infection with *Brachyspira* spp. since the intestinal and caecal microbiota can differ widely based on the conditions under which the mice are initially raised [11]. However, both mouse strains examined here were from the same source and showed similar faecal shedding patterns, suggesting that they are equally susceptible to infection. To further complicate the interpretation, some control mice (most evident in Experiment 2), developed catarrhal inflammation in the large intestines. While feeding TD85420 diet may increase the probably of developing this lesion, it was also observed a non-inoculated control mouse in Experiment 1 fed RMH diet. In any case, the severity of pathological lesions, particularly the presence of epithelial regeneration and neutrophilic inflammation in Experiment 2 were poorly associated with clinical signs.

## Conclusions

This research provided a number of important conclusions. Firstly, CF-1 mice appear to be more susceptible than C3H mice, and feeding TD85420 yielded the more consistent clinical signs following “*Brachyspira hampsonii*” clade II inoculation than feeding RMH3000 chow. Although not previously reported, assessing changes in faecal consistency provided a valuable and relevant measure of clinical enteric disease, with mild to severe soft, mucoid faeces commonly observed for lengthy periods (>7 days) in TD85420-fed inoculated mice. However, mice with high mean fecal scores did not all have severe lesions in large intestine, indicating pathology alone is insufficient to characterize enteric outcome following *Brachyspira* challenge in mice.

## Additional files

Additional file 1: Scoring template used to grade faecal consistency twice daily in experimentally inoculated mice. Experiment 1 scores: 0 = normal, formed faecal pellet, 1 = formed pellet with small mucous tail, 2) soft mucoid faeces, 3) faeces with blood (+/− mucus). For Experiment 2, score 2 was subdivided: slightly soft and mucoid faeces were scored as 2.0, whereas very soft and mucoid faeces were scored 2.5. Dark faeces (score 0, 1) are from mice fed RMH diet. Lighter faeces (score 2. 2.5, 3) are from mice fed TD85420. (TIFF 1522 kb)

Additional file 2: Scoring matrix for catarrhal inflammation: 0 = no notable changes; 1 = occasional sloughed epithelial cells on the surface or in the lumen (*arrow*); 2 = focal to multifocal groups of sloughed cells on the surface or in the lumen (*arrow*); 3 = one or more layers of sloughed cells covering more than 30% of the surface or multifocally >3 layers of sloughed cells on the surface or in the lumen (*arrow*). Magnification 40×. (TIFF 1522 kb)

Additional file 3: Scoring matrix for mucoid exudate: 0 = no notable changes; 1 = only notable in crypts or occasional strands in the lumen (*arrow*); 2 = frequent strands in the lumen (*arrow*); 3 = large lakes of mucus in the lumen (*arrow*). Magnification 40×. (TIFF 1522 kb)

Additional file 4: Scoring matrix for neutrophilic inflammation: 0 = no notable changes; 1 = occasional in the laminar propria (*arrow*) and/or submucosa; 2 = frequent in the laminar propria and/or submucosa (*arrow*); 3 = frequent in the laminar propria and/or submucosa and at the same time present in groups in the crypts and/or lumen (*arrow*). Magnification 40×. (TIFF 1522 kb)

Additional file 5: Scoring matrix for epithelial regeneration: 0 = no notable changes, 1 = thickened mucosa with frequent mitotic figures (*arrows*). Magnification 40×. (TIFF 1522 kb)

Additional file 6: Heat map displaying clinical signs and faecal shedding for Experiment 1 during the post-inoculation period. Culture positive results are represented by “+” on 5, 7, 9, 12, and 14 dpi (all samples were strongly β-haemolytic). Colour of square (*grey scale*) represents the faecal consistency score on the given day. Culture and PCR performed on the last faecal sample and colonic tissue collected at termination to confirm *Brachyspira* species of inoculation are indicated by *. (TIFF 1522 kb)

Additional file 7: Kaplan Meier survival curves showing number of healthy mice remaining in each group over time. Each incremental decrease represents at least one mouse developing soft, mucoid faeces (score 2). a Experiment 1 results indicate that mice fed TD85420 are more susceptible than RHM fed mice to developing soft, mucoid faeces following *“B. hampsonii”* or *B. hyodysenteriae* challenge. b Experiment 2 results indicate CF-1 mice are more susceptible than C3H mice to developing soft, mucoid faeces following *“B. hampsonii”* challenge. (TIFF 1522 kb)

Additional file 8: Heat map displaying clinical signs and faecal shedding in Experiment 2 during the post-inoculation period. Culture positive results are represented by “+” on 5, 7, 9 and 12 dpi, and termination (all samples were strongly β-haemolytic). Colour of square (grey scale) represents the faecal consistency score on the given day. Culture and PCR performed on the last faecal sample and colonic tissue collected at termination to confirm “*Brachyspira hampsonii”* are indicated by *. (TIFF 1522 kb)

## Abbreviations

Bhamp: “*Brachyspira hampsonii*”
Bhyo: *Brachyspira hyodysenteriae*
C3H: Charles River C3H mouse strain
CF-1: CF-1 Charles River mouse strain
GE: Genomic equivalents
H and E: Haematoxylin and eosin stain
RMH: RMH 3000 mouse chow
SD: Swine dysentery
Spp: Species
std. dev: Standard deviation
TD: TD85420 low zinc diet
