# Supplementary figures and images for: An optimized swine dysentery murine model to characterize shedding and clinical disease associated with “Brachyspira hampsonii” infection
Source: BMC Vet Res. 2017 Aug 22;13:261. doi: 10.1186/s12917-017-1166-5 (PMC5568335; doi:10.1186/s12917-017-1166-5)

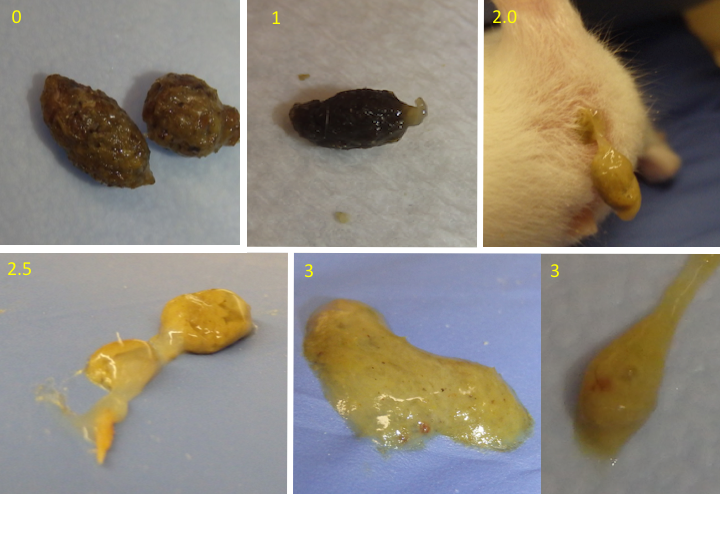

Supplement: Supplementary file 1 — Scoring template used to grade faecal consistency twice daily in experimentally inoculated mice. Experiment 1 scores: 0 = normal, formed faecal pellet, 1 = formed pellet with small mucous tail, 2) soft mucoid faeces, 3) faeces with blood (+/− mucus). For Experiment 2, score 2 was subdivided: slightly soft and mucoid faeces were scored as 2.0, whereas very soft and mucoid faeces were scored 2.5. Dark faeces (score 0, 1) are from mice fed RMH diet. Lighter faeces (score 2. 2.5, 3) are from mice fed TD85420. (TIFF 1522 kb) [file 12917_2017_1166_MOESM1_ESM.tiff]

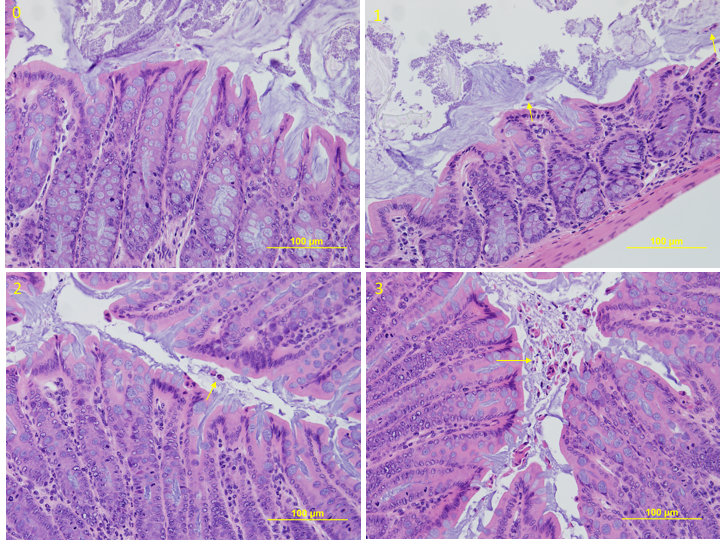

Supplement: Supplementary file 2 — Scoring matrix for catarrhal inflammation: 0 = no notable changes; 1 = occasional sloughed epithelial cells on the surface or in the lumen (arrow); 2 = focal to multifocal groups of sloughed cells on the surface or in the lumen (arrow); 3 = one or more layers of sloughed cells covering more than 30% of the surface or multifocally >3 layers of sloughed cells on the surface or in the lumen (arrow). Magnification 40×. (TIFF 1522 kb) [file 12917_2017_1166_MOESM2_ESM.tiff]

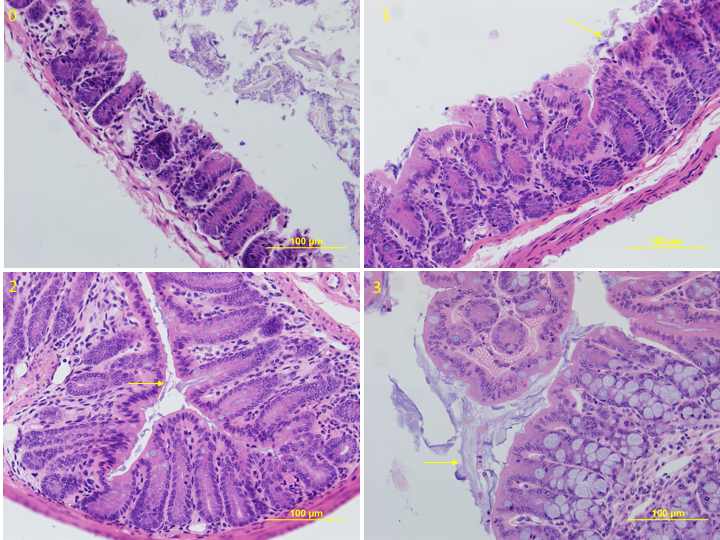

Supplement: Supplementary file 3 — Scoring matrix for mucoid exudate: 0 = no notable changes; 1 = only notable in crypts or occasional strands in the lumen (arrow); 2 = frequent strands in the lumen (arrow); 3 = large lakes of mucus in the lumen (arrow). Magnification 40×. (TIFF 1522 kb) [file 12917_2017_1166_MOESM3_ESM.tiff]

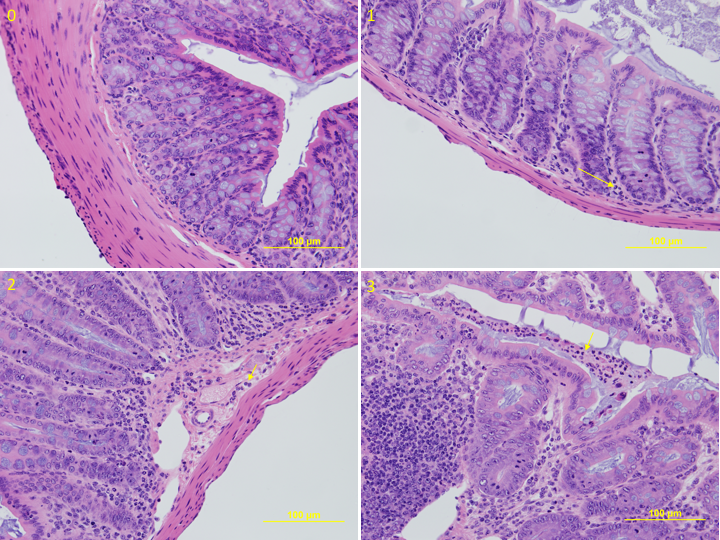

Supplement: Supplementary file 4 — Scoring matrix for neutrophilic inflammation: 0 = no notable changes; 1 = occasional in the laminar propria (arrow) and/or submucosa; 2 = frequent in the laminar propria and/or submucosa (arrow); 3 = frequent in the laminar propria and/or submucosa and at the same time present in groups in the crypts and/or lumen (arrow). Magnification 40×. (TIFF 1522 kb) [file 12917_2017_1166_MOESM4_ESM.tiff]

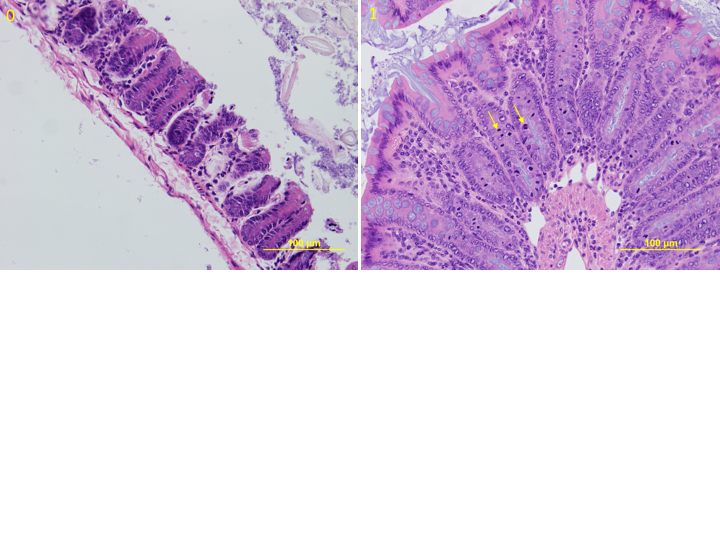

Supplement: Supplementary file 5 — Scoring matrix for epithelial regeneration: 0 = no notable changes, 1 = thickened mucosa with frequent mitotic figures (arrows). Magnification 40×. (TIFF 1522 kb) [file 12917_2017_1166_MOESM5_ESM.tiff]

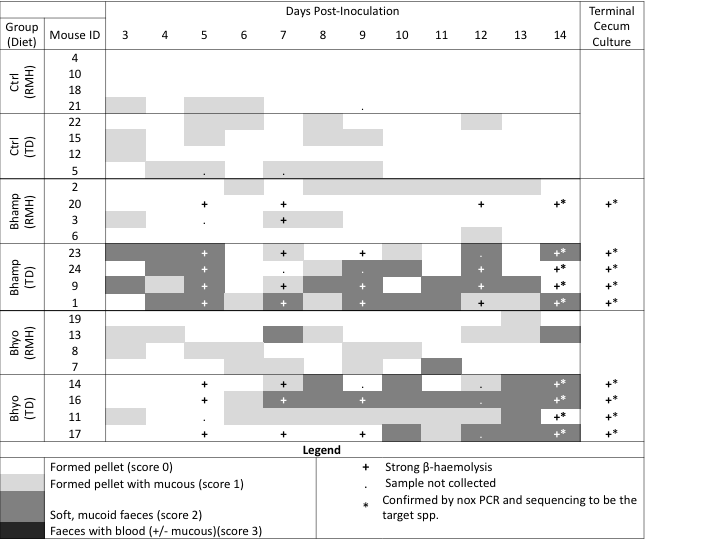

Supplement: Supplementary file 6 — Heat map displaying clinical signs and faecal shedding for Experiment 1 during the post-inoculation period. Culture positive results are represented by “+” on 5, 7, 9, 12, and 14 dpi (all samples were strongly β-haemolytic). Colour of square (grey scale) represents the faecal consistency score on the given day. Culture and PCR performed on the last faecal sample and colonic tissue collected at termination to confirm Brachyspira species of inoculation are indicated by *. (TIFF 1522 kb) [file 12917_2017_1166_MOESM6_ESM.tiff]

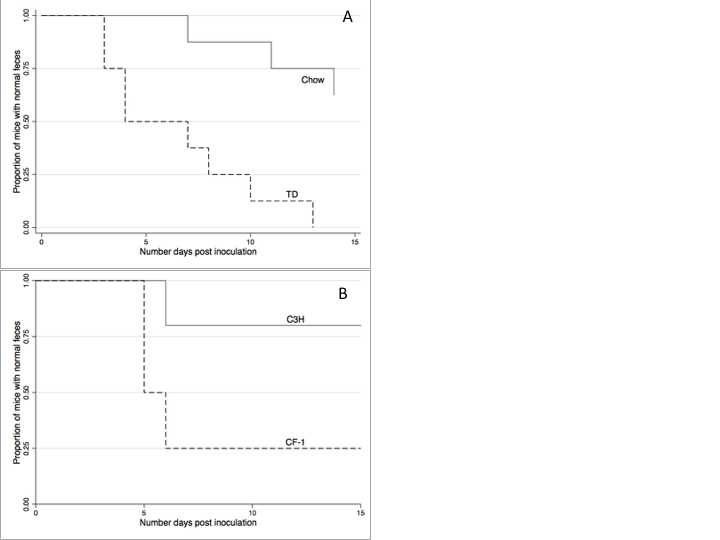

Supplement: Supplementary file 7 — Kaplan Meier survival curves showing number of healthy mice remaining in each group over time. Each incremental decrease represents at least one mouse developing soft, mucoid faeces (score 2). a Experiment 1 results indicate that mice fed TD85420 are more susceptible than RHM fed mice to developing soft, mucoid faeces following “B. hampsonii” or B. hyodysenteriae challenge. b Experiment 2 results indicate CF-1 mice are more susceptible than C3H mice to developing soft, mucoid faeces following “B. hampsonii” challenge. (TIFF 1522 kb) [file 12917_2017_1166_MOESM7_ESM.tiff]

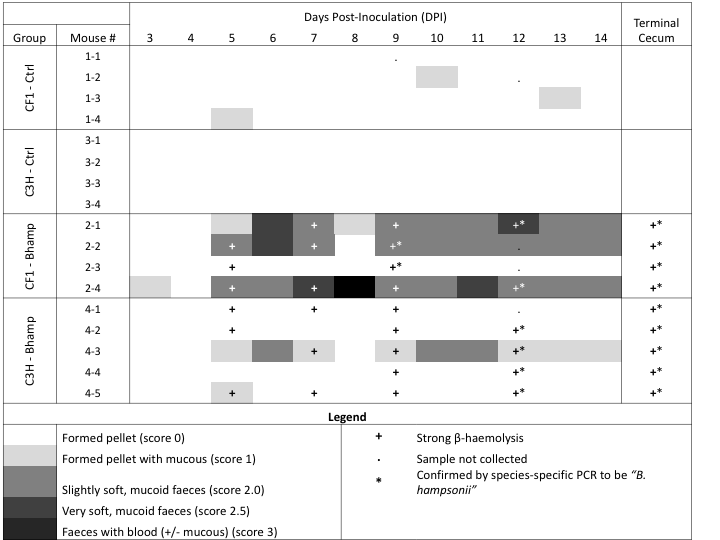

Supplement: Supplementary file 8 — Heat map displaying clinical signs and faecal shedding in Experiment 2 during the post-inoculation period. Culture positive results are represented by “+” on 5, 7, 9 and 12 dpi, and termination (all samples were strongly β-haemolytic). Colour of square (grey scale) represents the faecal consistency score on the given day. Culture and PCR performed on the last faecal sample and colonic tissue collected at termination to confirm “Brachyspira hampsonii” are indicated by *. (TIFF 1522 kb) [file 12917_2017_1166_MOESM8_ESM.tiff]
